# WSTF-associated regulation of GLYCTK and metabolic adaptation in colorectal cancer

**DOI:** 10.3389/fimmu.2026.1819148

**Published:** 2026-04-23

**Authors:** Liming Zhou, Yan Liu, Yufeng Li, Shasha Zheng, Jimin Guo, Shuai Chen, Yuan Yu, Wei Fang, Jingwu Li

**Affiliations:** 1 College of Life Sciences, North China University of Science and Technology, Tangshan, China; 2 The Cancer Institute, Hebei Key Laboratory of Molecular Oncology, Tangshan People’s Hospital, Tangshan, China

**Keywords:** colorectal cancer, GLYCTK, immune microenvironment, metabolic reprogramming, transcriptional regulation, WSTF

## Abstract

**Background:**

Colorectal cancer (CRC) is a leading cause of cancer-related morbidity and mortality worldwide, driven by complex genetic and epigenetic factors. The Williams syndrome transcription factor (WSTF/BAZ1B), an emerging oncogenic factor, has been implicated in chromatin remodeling and genome stability. However, the precise mechanisms in CRC progression, particularly the metabolic reprogramming processes, remain unclear.

**Methods:**

This study integrated transcriptomic, metabolomic, genome-wide ChIP-seq and summary-data-based Mendelian randomization (SMR) analyses in CRC cells and patient cohorts to study the WSTF regulatory network. WSTF knockdown and overexpression cell lines were combined with RNA-seq, LC-MS metabolomics and ChIP-seq annotation to identify direct targets and affected pathways. Multi-omics SMR using CRC GWAS, eQTL and mQTL datasets, together with bulk and single-cell RNA-seq, immune infiltration, functional assays and molecular docking were applied to explore GLYCTK as a candidate downstream effector of WSTF and a putative CRC-associated marker.

**Results:**

WSTF knockdown reprogrammed transcription and metabolism, activating glucose and stress-response pathways while suppressing ribosome biogenesis, genetic information processing and MYC-related programs. ChIP-seq suggested that WSTF mainly binds to promoter-proximal, GC-rich motifs and identified GLYCTK as a candidate downstream target whose expression is inversely associated with WSTF. SMR analysis suggested a methylation-dependent regulatory relationship in which GLYCTK expression is associated with CRC risk. The immune infiltration analyses suggested that GLYCTK is associated with an immune-cold tumor microenvironment, whereas functional assays indicated that GLYCTK promotes proliferation, migration and invasion in CRC cells. Drug screening identified phenylbiguanide and hydroxyfasudil as candidate compounds targeting GLYCTK-associated networks.

**Conclusions:**

WSTF may contribute to CRC progression through transcriptional and epigenetic-associated regulation of metabolic adaptation, while GLYCTK may represent a context-dependent downstream factor associated with CRC susceptibility and development.

## Introduction

Colorectal cancer (CRC) is one of the leading causes of cancer-related death worldwide, arising from complex genetic and epigenetic dysregulation (1). Williams syndrome transcription factor (WSTF), also known as bromodomain adjacent to zinc finger domain 1B (BAZ1B), is a chromatin-associated regulatory protein involved in chromatin remodeling, transcription, DNA replication and repair (2). WSTF is localized to chromosome 7q11.23 and was first recognized for its hemizygous deletions underlying Williams syndrome, a multisystem developmental disorder (3). More recent studies have discovered that WSTF is widely and dynamically expressed in diverse tissues and interacts with numerous chromatin-associated protein complexes. WSTF possesses a unique combination of functional structural domains including bromodomain (BrD), plant homeodomain (PHD) type zinc finger, and motifs involved in DNA binding and chromatin modification (3, 4). As a core subunit of the WICH (WSTF-ISWI chromatin-remodeling) and B-WICH complex, WSTF regulates nucleosome repositioning to facilitate DNA replication, transcription and repair (5, 6). The WICH complex is essential for promoting chromatin accessibility and ensuring faithful genome duplication during DNA replication (7). While the B-WICH complex critically regulates RNA polymerase I, II, and III-mediated transcription through mechanisms involving chromatin compaction and histone acetylation (8, 9).

A unique feature of WSTF is its kinase activity, through which it modulates the DNA damage response by the direct phosphorylation of histone H2AX on tyrosine 142 (10, 11). Moreover, recent reports have revealed that WSTF also phosphorylates DNA damage markers and modulates PCNA ubiquitination during DNA repair through direct association with ATAD5, providing another protection mechanism of genome stability under replication stress (12, 13). This modification integrates signaling pathways controlling DNA repair and apoptosis, indicating WSTF’s critical roles in genome maintenance and cellular fate determination.

Accumulating evidence implicates WSTF as an oncogenic factor in various cancers. Compared to normal tissues, WSTF expression is elevated in breast, lung and cervical cancers, where it promotes tumor cell proliferation, invasion and migration, mainly via activation of the PI3K/Akt and IL-6/STAT3 signaling pathways (14–16). Post-translational modifications, including acetylation and phosphorylation, appear to enhance the oncogenic activity of WSTF, underscoring its potential as a biomarker and therapeutic target (17). Integrative multi-omics analyses in this study suggested a potential association between WSTF and D-glycerate kinase (GLYCTK). This enzyme catalyzes the phosphorylation of D-glycerate, an important step in both fructose and serine metabolism (18, 19).

**Figure 1:**
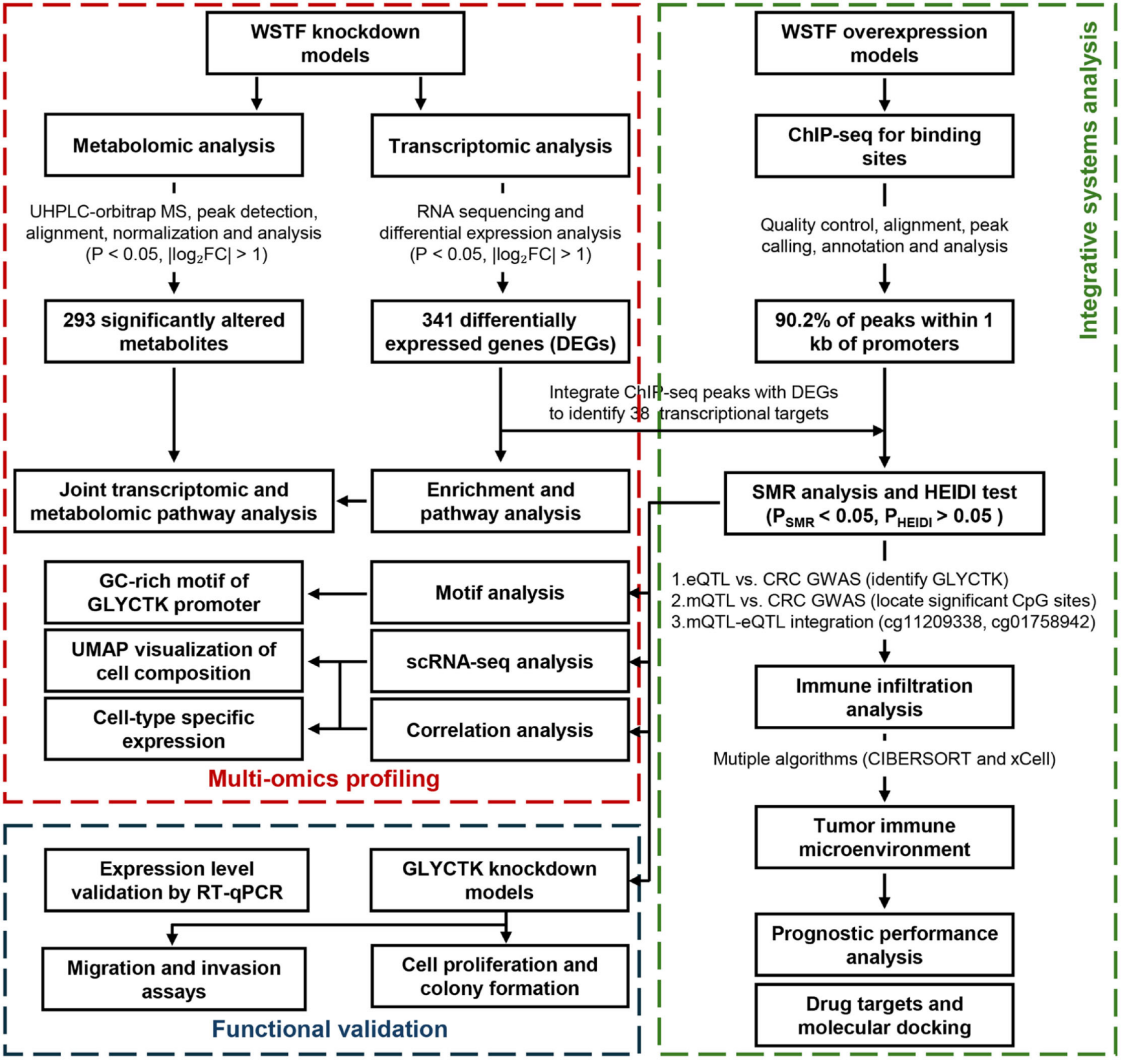
Workflow diagram.

Despite growing evidence linking WSTF to cancer biology, its molecular mechanisms in promoting CRC progression, particularly in tumor metabolic reprogramming, remain unclear. Thus, this study employed integrative multi-omics methods, such as transcriptomic profiling, metabolomic analysis, genome-wide ChIP-seq and Mendelian randomization, to investigate the potential association between WSTF and GLYCTK (**Figure 1**). WSTF modulates key transcriptional programs and metabolic pathways essential for maintaining tumor growth. While GLYCTK may act as a context-dependent mediator whose expression is associated with CRC susceptibility and tumor progression. Functional assays further indicate that GLYCTK influences proliferation and migration in CRC cells. Notably, a context-dependent model may help reconcile the apparent dual role of GLYCTK. In premalignant or non-malignant settings, GLYCTK may be linked to reduced CRC susceptibility, whereas in established tumors it may support metabolic adaptation and malignant progression. Therefore, we explored GLYCTK in both risk-associated and functional contexts to better understand its role across CRC development. These findings provide insight into WSTF-associated tumor metabolism and suggest that the WSTF-GLYCTK axis may represent a potential therapeutic target in colorectal cancer.

## Methods

### Cell lines and culture conditions

Normal human colon mucosal epithelial cells NCM460 and colorectal cancer cell lines SW480, SW620 and HT29 were used in this study. The cell lines present in this study were obtained from Procell Life Science & Technology Co., Ltd. (Procell, China). All cell lines were cultured in DMEM supplemented with 10% FBS at 37 °C in a humidified incubator with 5% CO2, and were passaged three times after thawing before use in experiments.

### WSTF knockdown and overexpression constructs in SW620 cells

WSTF knockdown was achieved by transfecting cells with specific siRNAs using Lipofectamine 2000 (Thermo Fisher Scientific) according to the manufacturer’s instructions. On the other hand, WSTF cDNA was cloned into an expression vector and introduced into SW620 cells using the same transfection reagent. Transfection efficiency and modulation of WSTF expression were validated by quantitative PCR and western blotting, and cells were collected for subsequent assays 48 h after transfection.

### Transcriptomic analysis of WSTF knockdown in SW620 cells

Transcriptomic analysis was conducted using RNA samples from SW620 cells transfected with si-NC (negative control) and si-WSTF (WSTF knockdown). Differentially expressed genes were identified by DESeq2 (20). DEGs were selected based on fold change and significance thresholds. Gene ontology enrichment analysis was performed to characterize DEGs function, using a hypergeometric test (Fisher’s exact test) to evaluate enrichment significance (21). KEGG pathway analysis identified significantly enriched pathways through a similar statistical approach (22). Furthermore, gene set enrichment analysis was applied to determine whether predefined gene sets exhibited statistically significant and consistent expression differences between si-NC and si-WSTF groups, involving calculation of enrichment scores, significance estimation, and multiple testing correction (23).

### Metabolomics analysis of WSTF knockdown in SW620 cells

si-WSTF or si-NC SW620 cells were harvested 48 hours later. Metabolites were extracted using organic solvents, the extracts were filtered and stored at -80 °C prior to LC-MS analysis. Metabolic profiling was performed using a Dionex Ultimate 3000 RS UHPLC system coupled with a Q-Exactive Orbitrap mass spectrometer (Thermo Fisher Scientific, USA) in both positive and negative ESI modes. Chromatographic separation was achieved with an ACQUITY UPLC HSS T3 column (2.1 × 100 mm, 1.8 μm, 45 °C) and a binary gradient of 0.1% formic acid in water and acetonitrile at a flow rate of 0.35 mL/min. Raw data were processed with Progenesis QI v2.3 for peak detection, alignment, and normalization. Metabolite identification was based on accurate mass, MS/MS spectra, and database searches.

### ChIP-seq analysis

ChIP-seq was performed on SW620 cells overexpressing WSTF. Cells were crosslinked with 1% formaldehyde, lysed, and chromatin was sonicated to 200–500 bp fragments. WSTF-bound chromatin was immunoprecipitated with specific antibody-conjugated magnetic beads, and input samples were collected as controls. Following extensive washes, DNA from both IP and input was purified after reverse crosslinking. Sequencing libraries were prepared using end-repair, A-tailing, adaptor ligation, PCR amplification, and size-selection (100–300 bp). Qualified libraries were sequenced on the Illumina HiSeq platform. Raw sequencing reads were processed using Cutadapt to remove adaptors, and Trimmomatic for quality filtering (24). Clean reads were assessed using FastQC and aligned to the human reference genome (hg38) using Bowtie2 (25, 26). Duplicates were removed, and high-quality uniquely mapped reads were retained for analysis. Peak calling was performed with MACS2, using the input DNA as a control (27). Peaks were annotated with ChIPseeker, and motif analysis was conducted with MEME (28, 29). Visualization of enrichment and peak distribution was performed using Deeptools and Circos (30, 31).

### Summary-data-based Mendelian randomization analysis

Genome-wide association study (GWAS) summary statistics for colorectal cancer (CRC) were obtained from the FinnGen research project (R11, https://www.finngen.fi/en/access_results), encompassing 8,801 CRC cases and 345,118 controls of European ancestry (32). Blood cis-eQTL summary statistics for GLYCTK were sourced from the eQTLGen consortium (http://www.eqtlgen.org), which includes 31,684 whole blood and PBMC samples (33). Blood cis-mQTL summary data for DNA methylation sites were derived from a European ancestry meta-analysis, combining the Brisbane Systems Genetics Study (BSGS, n = 614) and the Lothian Birth Cohorts (LBC, n = 1,366) (34). All datasets underwent quality control and imputation to ensure high-quality analysis.

Three-step SMR approach was performed as described previously (34). First, SMR analysis was conducted between GLYCTK cis-eQTLs and CRC GWAS to assess the putative causal effect of gene expression on CRC. Second, cis-mQTLs of methylation probes within ±0.5 Mb of GLYCTK were analyzed against CRC GWAS to identify regulatory CpG sites impacting risk. Finally, significant methylation probes from step two were further tested for causal relationships with GLYCTK expression using mQTL-eQTL SMR integration. All analyses were performed using the SMR software (https://cnsgenomics.com/software/smr/) developed by the Yang Lab, with default quality control and parameter settings. Only signals passing both SMR and HEIDI criteria (pSMR < 0.05, pHEIDI > 0.05) were considered robust.

### Immune infiltration analysis

Immune cell infiltration within the tumor microenvironment was inferred from TCGA bulk RNA-sequencing data through transcriptome deconvolution methods. Normalized gene-level expression matrices were analyzed with several established algorithms (CIBERSORT and xCell), primarily via the immunedeconv package (35). Regression-based methods estimated relative cell fractions by modeling bulk expression as weighted combinations of immune cell-specific signatures, whereas enrichment-based methods computed normalized abundance scores for predefined immune and stromal gene sets. The resulting infiltration profiles were used in downstream analyses to quantify associations between gene expression and specific immune cells.

### Single cell transcriptome analysis

Single-cell RNA sequencing (scRNA-seq) data were obtained from the gene expression omnibus (GEO) under accession GSE200997, comprising colorectal cancer specimens from 16 patients and matched adjacent normal tissues. Raw sequence data were processed using the Seurat package (v5.2.1). Cells meeting the following criteria were retained: gene number >500 and mitochondrial gene percentage <25%. The potential doublets were detected and removed by scDblFinder. Data normalization, identification of highly variable genes (HVGs), principal component analysis (PCA) and batch correction were performed as previously (36). Integrated analysis corrected batch effects across samples, guided by canonical correlation analysis (CCA) leveraging the top 30 integrated dimensions. Dimensionality reduction was performed utilizing principal component analysis on highly variable genes, with the top 10 principal components informing uniform manifold approximation and projection (UMAP) embedding. Unsupervised graph-based clustering (resolution = 0.68) identified 17 distinct cell types, subsequently annotated using marker gene expression profiles and validated by differential expression analyses. Cluster identity was confirmed by heatmap visualization of top marker genes (FindAllMarkers).

### Drug prediction and molecular docking

Based on the median expression of GLYCTK/WSTF, patients were divided into high- and low-risk groups for survival analysis. Differential expression analysis between the two groups identified differentially expressed genes (DEGs). The DEGs were submitted to the connectivity map (CMap, https://clue.io) online platform to identify candidate compounds, with connectivity scores less than -70 selected as potential therapeutics for further analysis. Candidate drugs and small molecules from the previous screening were queried in the STITCH database (http://stitch.embl.de) to retrieve predicted target genes. Protein structures for these targets were downloaded from the RCSB protein data bank (https://www.rcsb.org/), and 3D structures of candidate compounds were obtained from the PubChem database (https://pubchem.ncbi.nlm.nih.gov/). Molecular docking was performed using AutoDock 4 (version 4.2.6), with binding free energies below -5 kcal/mol indicating stable binding. Visualization of docking results employed PyMOL (version 2.6.2) and Discovery Studio (version 4.5).

### GLYCTK stable knockdown

Three shRNA sequences targeting GLYCTK were designed using DSIR and siDirect. Complementary oligonucleotides were synthesized, annealed, and cloned into pSilencer-2.1-U6-neo vector. The constructs were transformed into XL1-Blue cells and selected with ampicillin. For stable knockdown, 3 × 10^5^ cells per well were seeded in 6-well plates, transfected with plasmid DNA using Lipofectamine 2000, and cultured. After 48 hours, cells were collected with G418 for 14 days, with medium refreshed every 48 hours. Single-cell clones were isolated by limiting dilution and expanded for further analysis.

### RNA extraction and quantitative real-time PCR

Total RNA was extracted using TRIzol reagent (Invitrogen) according to the manufacturer’s protocol. Genomic DNA was removed with gDNA Wiper Mix (Vazyme, China). Complementary DNA was synthesized using HiScript II Q RT SuperMix (Vazyme, China) at 50 °C for 15 min and 85 °C for 5 s. RT-qPCR was performed on a Thermal Cycler Dice Real-Time System (Takara, Japan) using SYBR Premix Ex Taq II (Takara, Japan) under the following conditions: 95 °C for 10 min, followed by 45 cycles of 95 °C for 30 s, 56 °C for 30 s and 72 °C for 30 s. β-actin served as the internal control.

### Western blot

Whole-cell lysates were prepared from HT29 control cells and GLYCTK-knockdown clones using RIPA buffer containing protease inhibitors. Protein concentrations were measured by BCA assay, and equal amounts of protein were denatured in loading buffer, boiled, and separated by SDS–PAGE, followed by transfer onto PVDF membranes. Membranes were blocked with 5% non-fat milk in TBST and incubated overnight at 4 °C with primary antibodies against GLYCTK and GAPDH. After incubation with HRP-conjugated secondary antibodies, signals were detected by enhanced chemiluminescence and imaged, and GLYCTK levels were quantified relative to GAPDH to evaluate knockdown efficiency.

### Cell viability assay (CCK-8)

To assess cell proliferation, 2 × 10^3^ cells per well were seeded in 96-well plates. After 24, 48, or 72 h, 10 µL CCK-8 reagent (Beyotime) was added to each well and incubated for 2 h. Absorbance at 450 nm was measured using a microplate reader (BioTek, USA).

### Transwell migration and invasion assays

To evaluate migratory and invasive capacities, transwell assays were performed using 8-µm pore inserts (Corning, USA). 3 × 10^4^cells in serum-free DMEM were seeded in the upper chamber, with invasion assays employing inserts pre-coated with a 1:5 dilution of Matrigel (BD Biosciences, USA) for 2 h at 37 °C. The lower chamber contained 600 µL DMEM supplemented with 20% FBS. After 24 h, non-migrating/invading cells were removed with a cotton swab, and membranes were fixed with methanol:glacial acetic acid (3:1) for 30 min, stained with 0.1% crystal violet for 15 min, and photographed under an inverted microscope (Olympus, Japan). Five random fields per insert were analyzed using ImageJ.

## Results

### Transcriptomic analysis of WSTF in colorectal cancer

**Figure 2:**
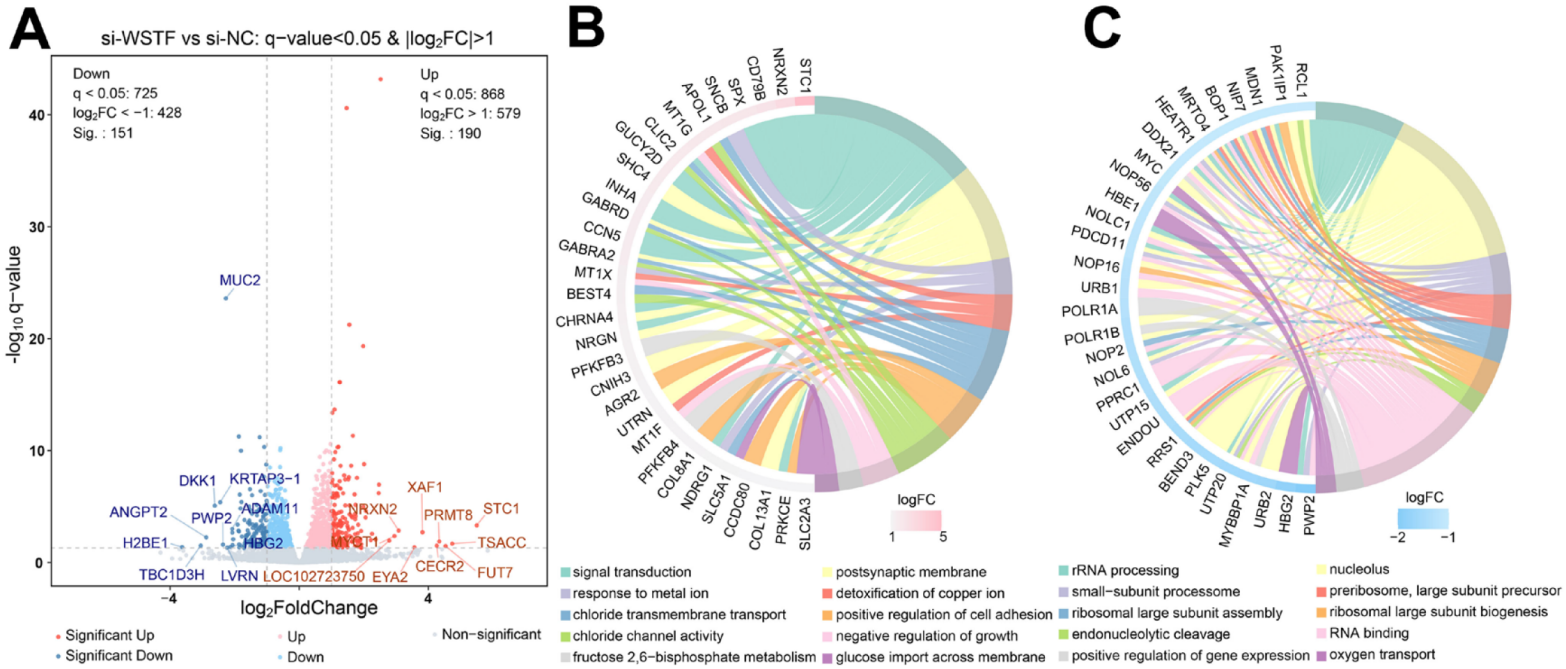
Transcriptomic analysis of WSTF knockdown in SW620 cells. **(A)** Volcano plot demonstrating DEGs between si-WSTF (WSTF knockdown) and si-NC (control) groups in SW620 cells. **(B)** Chord diagram of enrichment analysis for upregulated DEGs. The right half-circle represents the top ten enriched categories (with the smallest p-values), the left half-circle shows the top 10 genes with the highest |log_2_FC| within each category. **(C)** Chord diagram of enrichment analysis for downregulated DEGs.

To investigate the functional roles and regulatory mechanism, WSTF was silenced in SW620 CRC cells. Transcriptome sequencing was subsequently performed on this model, and differential expression analysis (p < 0.05, |log_2_FC| > 1) identified 341 differentially expressed genes (DEGs). These DEGs consisted of 190 upregulated and 151 downregulated genes (**Figure 2A**; **Supplementary Table 1**).

Gene ontology (GO) enrichment analysis demonstrated that upregulated DEGs are primarily involved in signal transduction, chloride ion transport and glucose metabolism, indicating that WSTF knockdown activates cellular stress responses and metabolic adaptation pathways (**Figure 2B**). In contrast, downregulated DEGs were significantly enriched in rRNA processing, ribosome biogenesis and regulation of gene expression, suggesting that WSTF supports cell proliferation and growth by maintaining ribosome assembly and protein synthesis capacity (**Figure 2C**).

**Figure 3:**
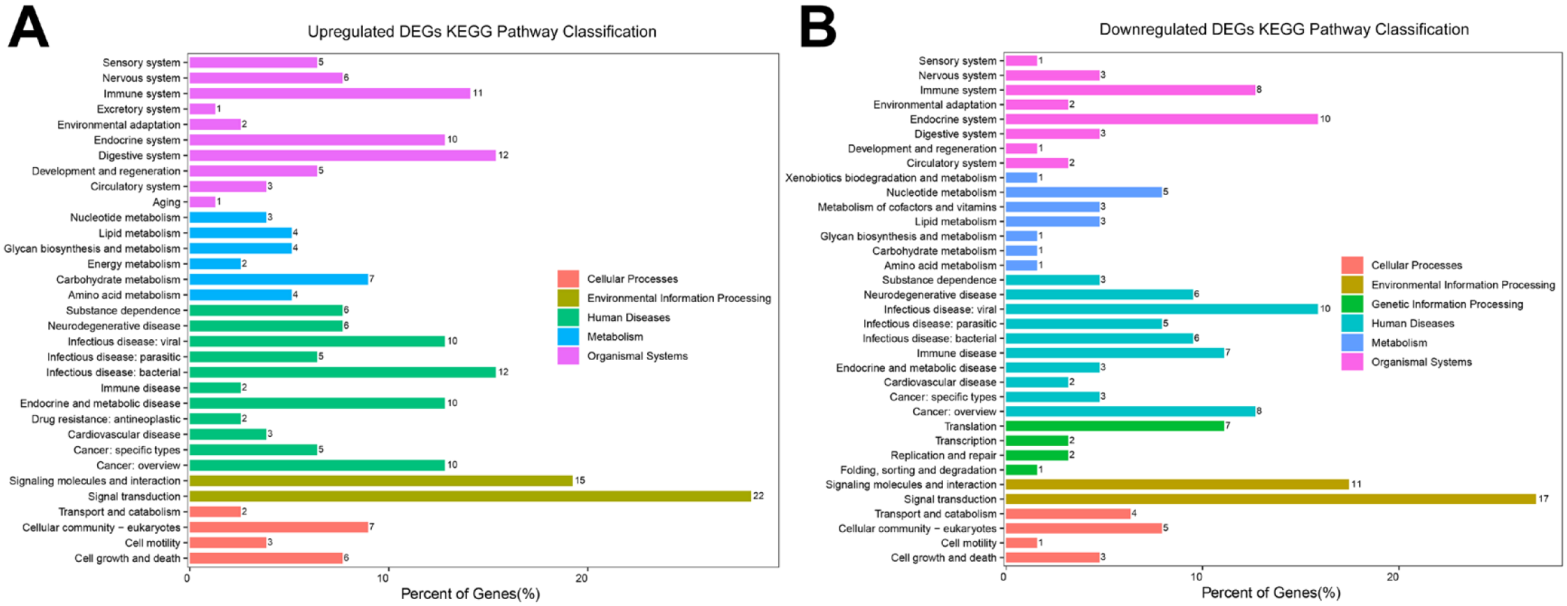
KEGG pathway analysis of DEGs. **(A)** Upregulated DEGs and **(B)** downregulated DEGs were categorized into distinct groups according to KEGG pathways. The bars represent the percentage of genes enriched to each pathway.

KEGG analysis showed that upregulated DEGs were mainly enriched in pathways related to signal transduction, cancer, carbohydrate metabolism, cell growth and death. In comparison, downregulated DEGs were primarily enriched in genetic information processing pathways (including translation, transcription, replication and repair), signal transduction, cancer and nucleotide metabolism (**Figure 3**). These findings are partially consistent with the results from the GO enrichment analysis.

**Figure 4:**
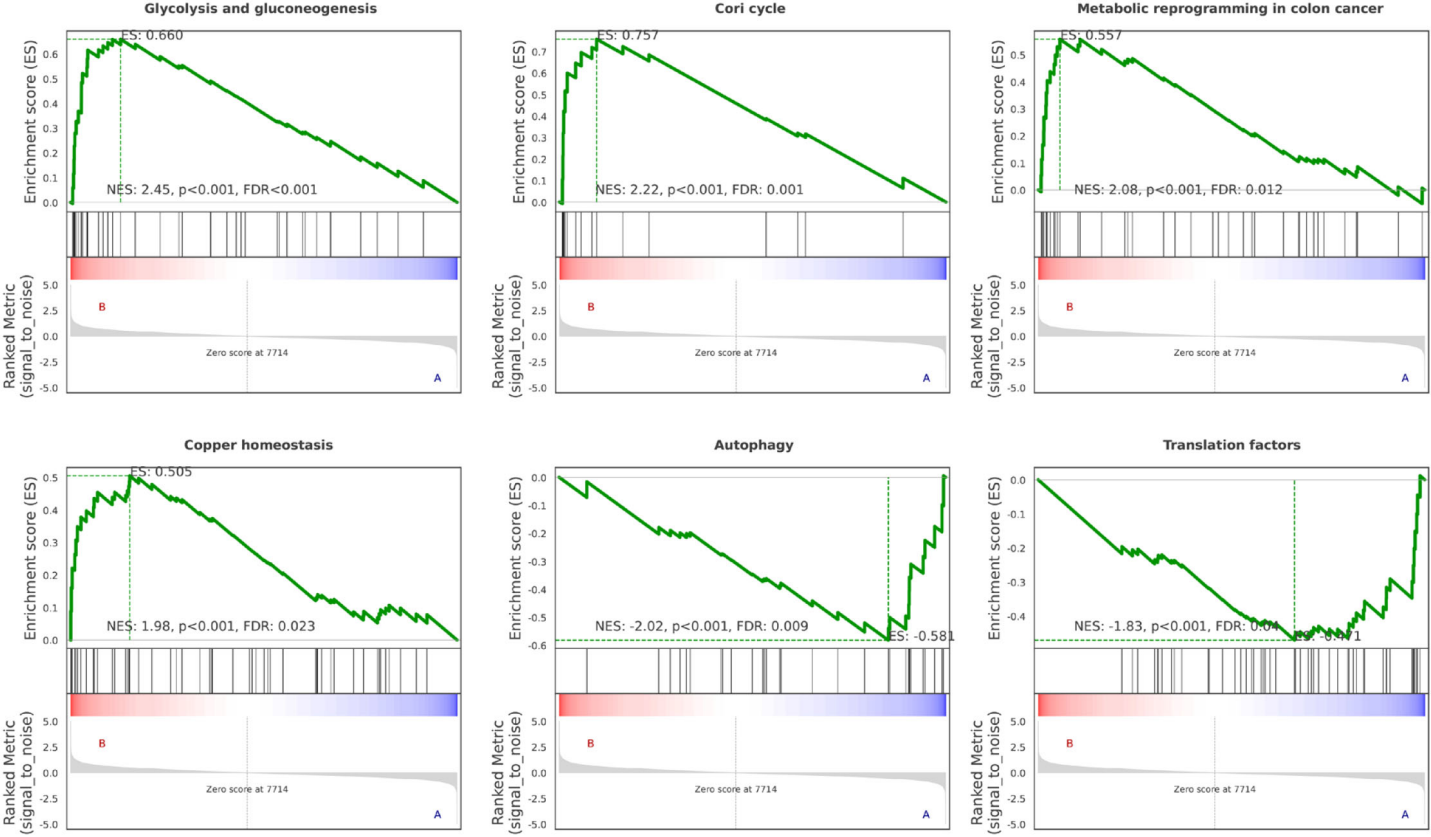
GSEA plots for six pathways. Pathways with high absolute normalized enrichment score (NES) and a false discovery rate (FDR) < 0.05 were selected. The enrichment score (ES) curve (green) reflects the degree to which each gene set is overrepresented at the top or bottom of the ranked gene list. The vertical black lines indicate the positions of pathway genes within the ranked list. The color bar shows the distribution of the ranked metric, with red and blue indicating up- and down-regulation, respectively.

Gene set enrichment analysis (GSEA) further demonstrated that knockdown of WSTF markedly promoted glycolysis (NES = 2.45, FDR < 0.001), Cori cycle (NES = 2.22, FDR = 0.001) and metabolic reprogramming in colon cancer (NES = 2.08, FDR = 0.012). Concurrently, it suppressed autophagy (NES = -2.02, FDR = 0.009) and translation factors (NES = -1.83, FDR = 0.04). These findings collectively suggest that WSTF is associated with tumor metabolic adaptation and cellular homeostasis (**Figure 4**).

### Metabolomic profiling of WSTF knockdown in colorectal cancer

**Figure 5:**
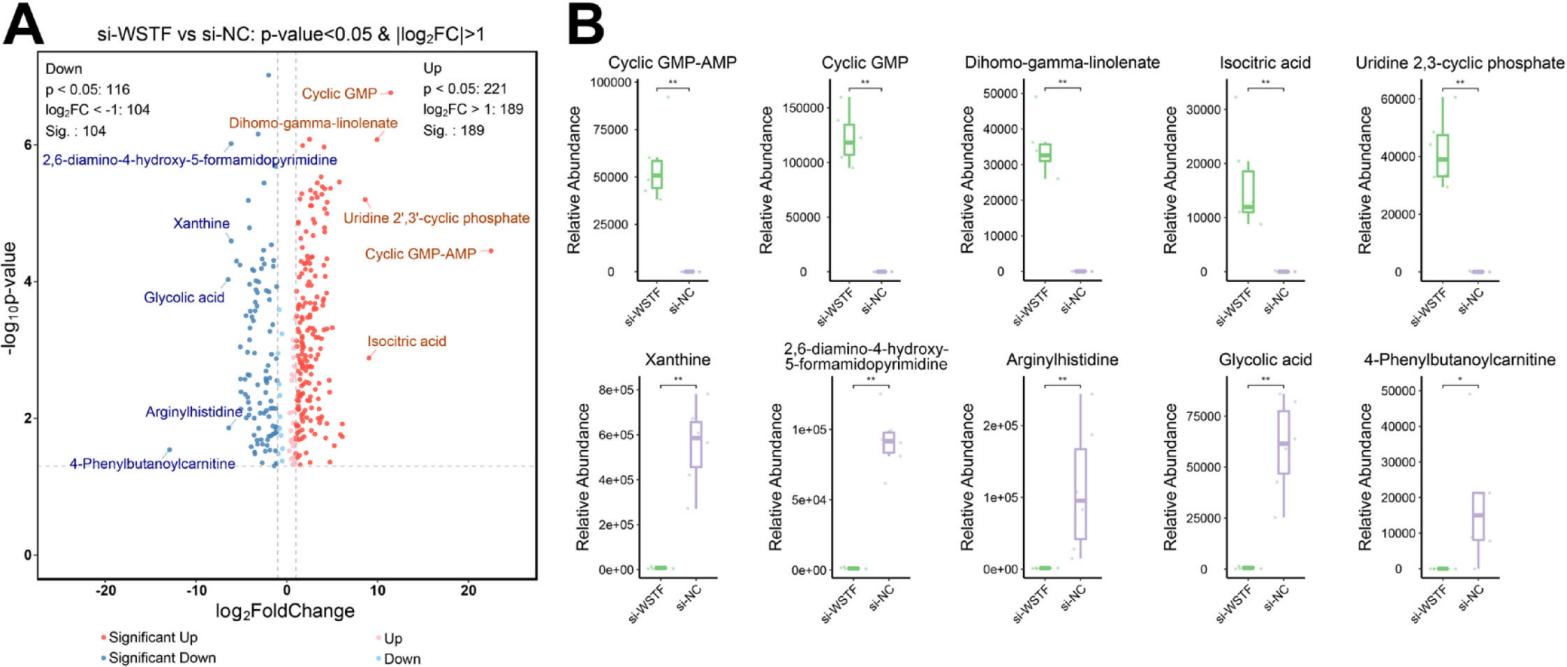
Metabolomic analysis of WSTF knockdown in SW620 cells. **(A)** Volcano plot depicting differentially abundant metabolites between si-WSTF (WSTF knockdown) and si-NC (control) groups (p < 0.05, |log_2_FC| > 1). The five most significantly upregulated and downregulated metabolites are labeled. **(B)** Box plots showing the relative abundance of the top five upregulated and downregulated metabolites in si-WSTF and si-NC groups. Each box represents the distribution across biological replicates, with statistical significance indicated (*p < 0.05, **p < 0.01).

To further clarify the metabolic reprogramming of WSTF knockdown cell lines, untargeted metabolomics profiling was performed in si-NC (control) and si-WSTF (WSTF knockdown) SW620 cell lines. Differential analysis identified 293 significantly altered metabolites (p < 0.05, |log_2_FC| > 1), with 189 upregulated and 104 downregulated in the si-WSTF group (**Figure 5A**; **Supplementary Table 2**). Among the most upregulated metabolites, Cyclic GMP-AMP and Cyclic GMP are central second messengers, mediating innate immune signaling and a variety of intracellular pathways, supporting the transcriptome-driven enrichment of nucleotide signaling and stress response pathways. Isocitric acid, a key tricarboxylic acid (TCA) cycle intermediate, mirrors GSEA results showing increased glycolysis and metabolic reprogramming. Conversely, the top downregulated metabolites provide additional mechanistic insight. Xanthine and 2,6-diamino-4-hydroxy-5-formamidopyrimidine, linked to purine catabolism and DNA damage repair, echo the suppression of genetic information processing genes identified in KEGG and GO analyses. The decrease in Glycolic acid reflects alterations in amino acid and energy metabolism, consistent with transcriptomic evidence of disrupted metabolic and translational homeostasis. Boxplots of these top metabolites further confirm substantial metabolic changes induced by WSTF knockdown (**Figure 5B**). Together, these data suggest that WSTF is associated with changes in nucleotide metabolism, energy balance and signaling transduction at both the transcriptional and metabolic levels.

### Genome-wide WSTF binding patterns and target genes in colorectal cancer

**Figure 6:**
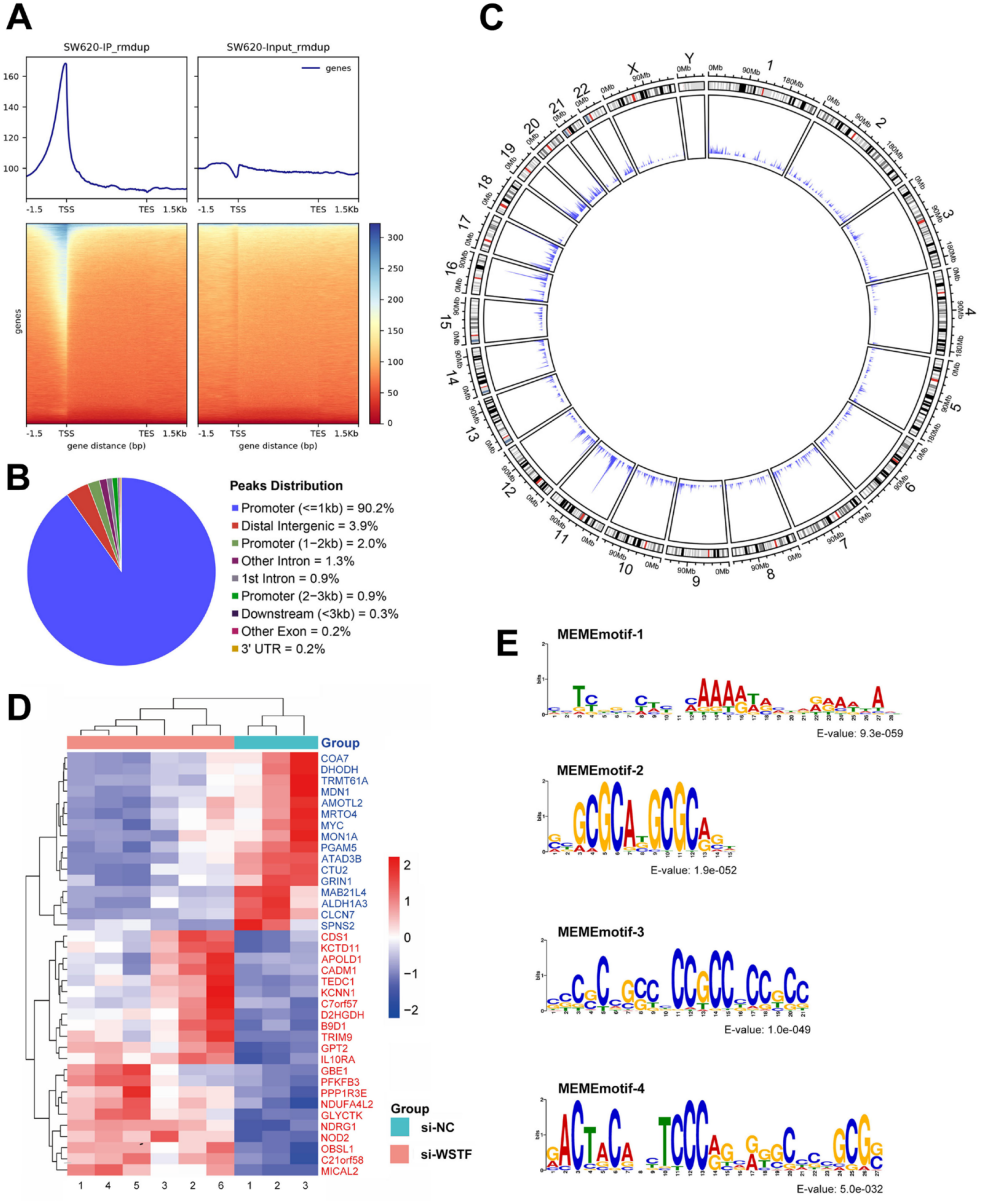
Genomic profiling of WSTF binding and its association with gene expression. **(A)** The distribution of ChIP-seq signals. A notable enrichment is observed at transcription start sites (TSS) in WSTF ChIP (IP) sample compared with input controls. **(B)** Pie chart of genomic distribution. **(C)** Chromosomal locations and density of WSTF binding peaks. The outer track shows chromosomal positions and the inner track represents binding density in 1-Mb bins. **(D)** Expression heatmap of 38 overlapping genes between DEGs and ChIP-seq annotated genes. **(E)** MEME motif analysis of WSTF ChIP-seq peaks.

Considering that WSTF functions in chromatin remodeling and transcriptional regulation, ChIP-seq analysis was performed to investigate its genome-wide binding profile in WSTF overexpression SW620 cells. As shown in **Figure 6A**, the gene body enrichment analysis demonstrated robust ChIP signal accumulation at transcription start sites (TSS) compared to the input (control), as visualized by deeptools and heatmaps. This enrichment around TSS regions is consistent with high-quality immunoprecipitation and suggests a promoter-proximal binding preference. Subsequently, Annotation of ChIP-seq peaks using ChIPseeker revealed that a substantial majority (90.2%) reside within proximal promoter regions (≤1 kb of TSS), while only a small fraction localizes to distal intergenic areas (3.9%) and other genomic features, such as introns and exons (**Figure 6B**; **Supplementary Table 3**). This distribution indicates a strong preference of WSTF for promoter proximal binding regions, consistent with a role in transcriptional regulation.

The circular genomic overview delineates the chromosomal landscape of WSTF binding, revealing a pattern of broad distribution across all chromosomes with sites of local enrichment (**Figure 6C**). This widespread distribution implicates WSTF in the extensive regulation of the genome. The intersection of DEGs and ChIP-seq annotated genes identified 38 overlapping candidate genes. Their expression patterns are displayed in the heatmap (**Figure 6D**).

After peak calling and annotation, the top 1000 peaks ranked by fold change were obtained, and genomic sequences (± 75 bp from the summit) were extracted for MEME motif analysis. The four top-ranked motifs by E-value are presented in **Figure 6E**. Motif 1 is defined by a central poly-adenine (AAAA) flanked by cytosines and thymines. Motifs 2 and 3 are characterized by GC-repeat clusters, indicating a GC-rich pattern. Motif 4 presents a more complex architecture with a prominent GC core and varied flanking nucleotides, suggesting diverse binding specificity. Collectively, these patterns exhibit the potential of WSTF to recognize both homotypic and mixed nucleotide sequences, aligning with its transcriptional regulatory function. Their significant enrichment (low E-values) confirms their biological relevance in WSTF-mediated gene regulation.

### SMR analysis of methylation-associated GLYCTK in colorectal cancer

**Figure 7:**
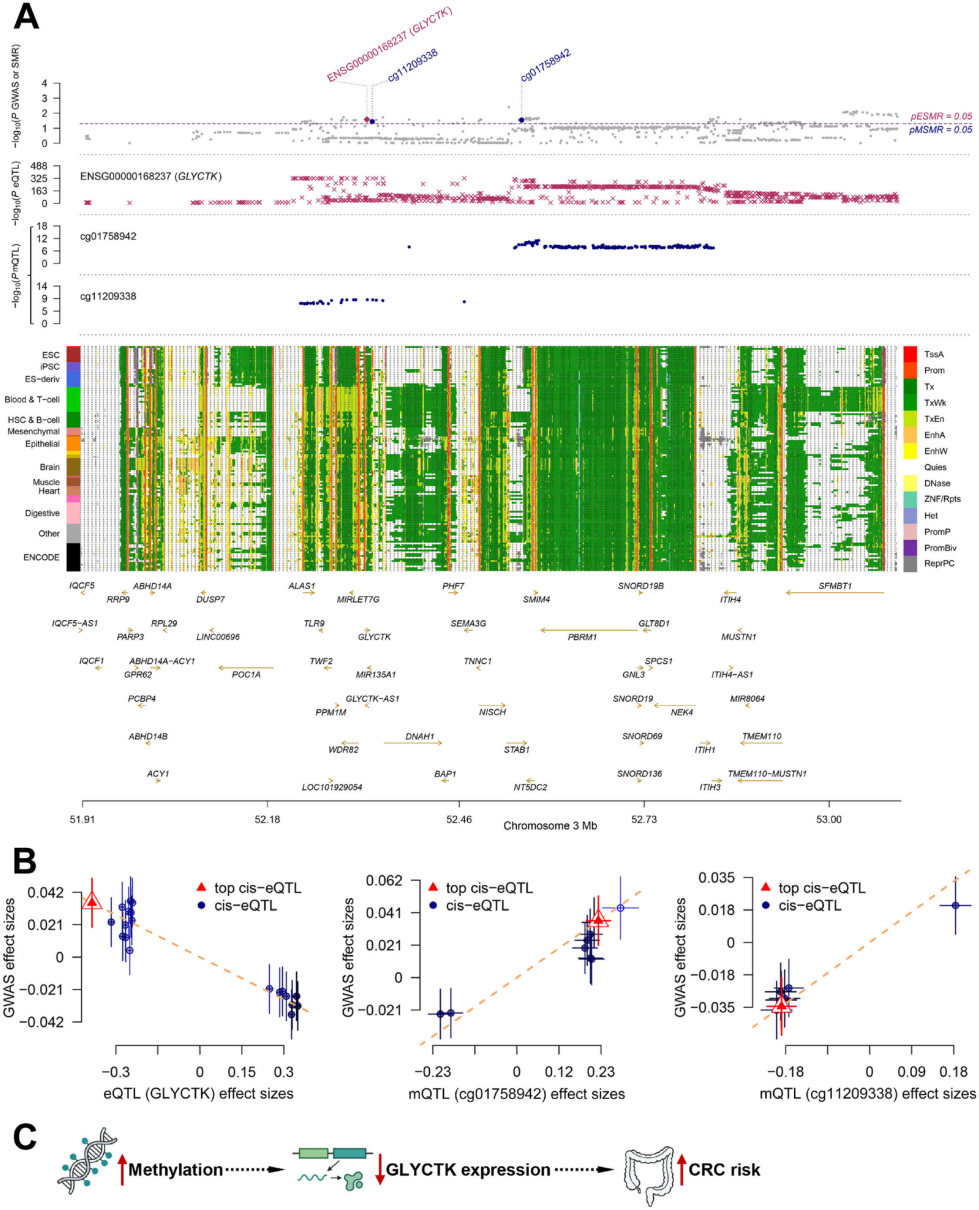
The SMR analysis of GLYCTK in colorectal cancer. **(A)** Regional association plots displaying genetic and epigenomic architecture at the GLYCTK locus on chromosome 3. The top panel shows GWAS association -log_10_(p) values for CRC susceptibility. The second panel presents -log_10_(p) values for cis-mQTLs. The third panel presents -log_10_(p) values for cis-eQTLs in the region. The fourth panel illustrates chromatin state annotations, color-coded by functional category. Gene locations and names are annotated along the X-axis. **(B)** Scatter plots for SMR results. Effect sizes between GLYCTK and CRC (left), effect sizes between two methylation sites and CRC (middle and right). **(C)** Overview of the SMR framework.

To reveal the causal mechanisms underlying candidate genes in colorectal cancer (CRC), we conducted a summary-data-based Mendelian randomization (SMR) analysis. Firstly, among 38 candidate genes identified by the intersection of differentially expressed genes and ChIP-seq annotation, only GLYCTK displayed a significant and specific association with CRC risk (pSMR < 0.05, pHEIDI > 0.05) according to SMR analysis of eQTL and CRC GWAS data (**Figure 7A**; **Supplementary Table 4**). This suggests a potential association between GLYCTK expression and CRC susceptibility. Secondly, within ±0.5 Mb of GLYCTK, SMR analysis of mQTL and CRC GWAS summary statistics identified 65 methylation sites, of which 9 showed significant evidence of a causal association with CRC (**Figure 7A**; **Supplementary Table 5**). These methylation sites potentially mediate genetic risk for CRC in proximity to GLYCTK. Finally, integrating mQTL and GLYCTK eQTL data, 2 methylation sites (cg11209338 and cg01758942) demonstrated strong causal relationships with GLYCTK expression (pSMR and pHEIDI were 1.6×10^-9^ & 0.077 and 2.7×10^-11^ & 0.087, respectively), indicating that GLYCTK transcription may be regulated by methylation at these sites (**Supplementary Table 6**).

Locus zooming plots illustrated strong co-localization of GWAS, eQTL, and mQTL signals near GLYCTK, and chromatin state annotation across various tissues highlighted the regulatory complexity of this locus. Three-step SMR analysis identified a coordinated regulatory model based on effect sizes (β). Methylation at cg11209338 and cg01758942 was negatively associated with GLYCTK expression and positively associated with CRC risk, whereas GLYCTK expression was negatively associated with CRC risk. Collectively, these findings suggest that methylation-mediated suppression of GLYCTK may contribute to CRC susceptibility (**Figures 7B, C**).

**Figure 8:**
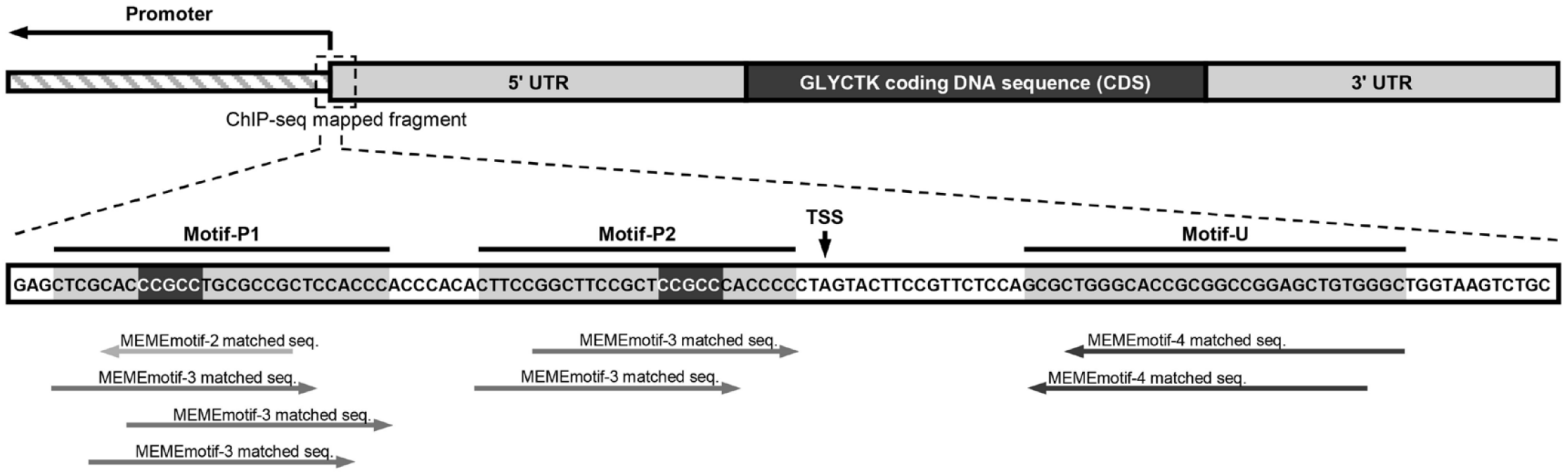
MEME motif analysis of ChIP-seq mapped fragments within the GLYCTK locus. The sequence enclosed by the dashed box corresponds to the ChIP-seq peak (SW620_peak_1988) located at the junction between the GLYCTK promoter and its 5’ UTR. Part of the sequence in this region is shown in an enlarged view, highlighting three distinct motif types: motif-P1 and motif-P2 both situated within the promoter region, and motif-U positioned in the 5’ UTR.

Motif analysis of GLYCTK regulatory region uncovered three spatially distinct clusters of WSTF binding motifs within the promoter and 5’ UTR (**Figure 8**). Two adjacent motifs (motif-P1 and motif-P2) in the promoter region shared overlapping sequences with GC-rich content and a conserved CCGCC core, indicative of a common WSTF recognition site. The repetitive GC pattern (CC/GC/CG dinucleotides) likely facilitates stable protein-DNA binding, which is consistent with the overall GC-rich characteristics identified in the genome-wide analysis. The third motif (motif-U) within the 5’ UTR contained multiple GC repeats and composite elements, which may facilitate the formation of stable secondary nucleic acid structures (such as stem-loops). Taken together, these motifs implicate diverse mechanisms through which WSTF may regulate GLYCTK expression. The promoter motifs likely serve as putative WSTF binding sites that modulate transcription initiation, whereas the 5’ UTR motif may mediate post-initiation regulation, supporting a hypothesis of GLYCTK transcriptional regulation by WSTF in colorectal cancer cells.

### Correlated dysregulation of WSTF and GLYCTK in colorectal cancer

**Figure 9:**
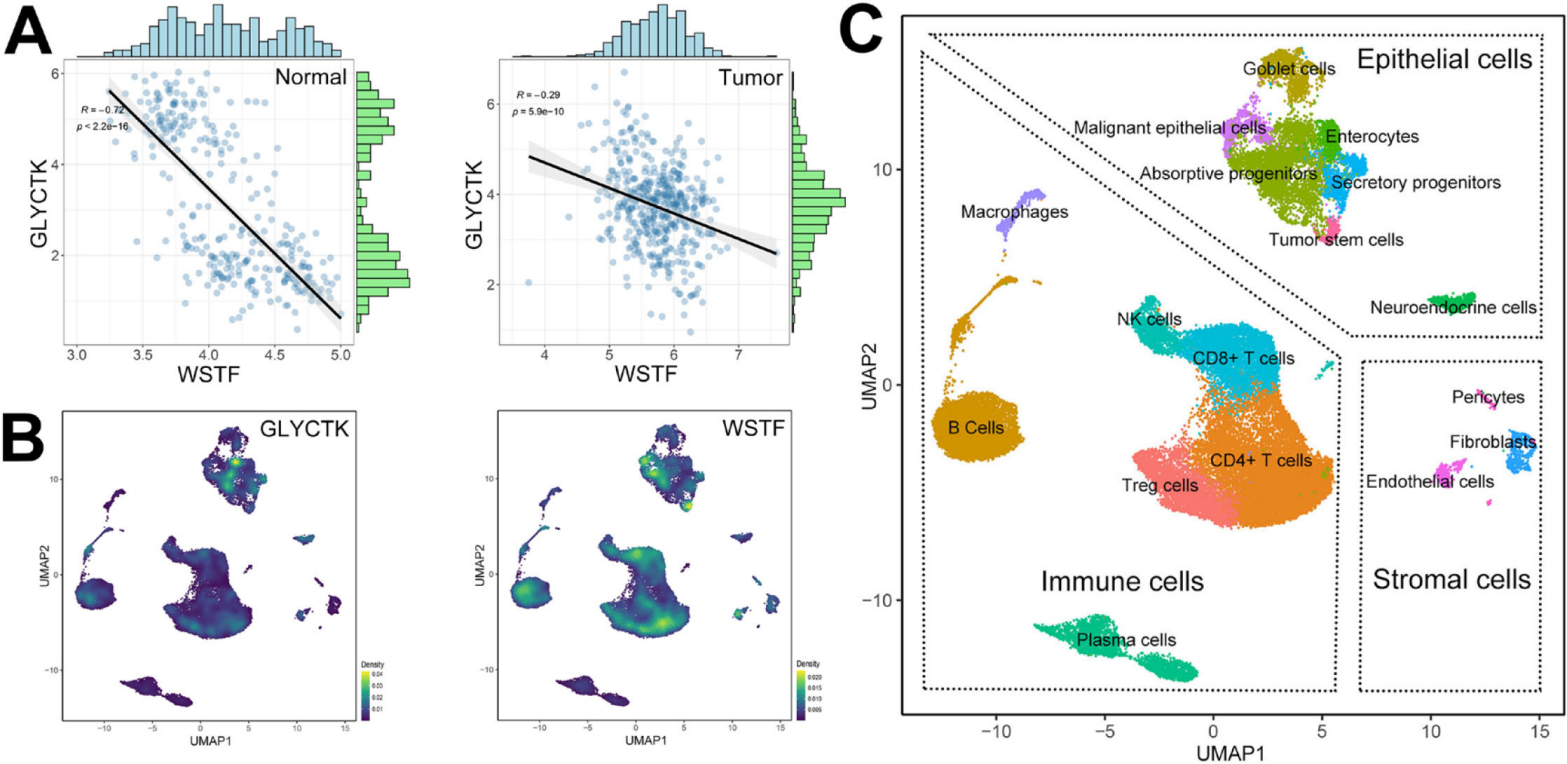
Integrated bulk and single-cell characterization of the WSTF-GLYCTK axis in colorectal cancer. **(A)** Correlation analysis between GLYCTK and WSTF expression in bulk transcriptomic data from normal tissues (left) and tumor (right). **(B)** Single-cell RNA sequencing density maps showing the distribution of GLYCTK (left) and WSTF (right) expression. **(C)** UMAP visualization of distinct cell clusters. Each cell type is color-coded as indicated in the image.

To further reveal the biological context of the WSTF-GLYCTK axis, we next examined their coordinated expression patterns in colorectal tissues. In paired bulk transcriptomic datasets, GLYCTK levels were inversely correlated with WSTF in normal colon tissue, suggesting an opposing expression pattern under physiological conditions. In contrast, this negative correlation was significantly attenuated in colorectal tumors (**Figure 9A**), suggesting that malignant transformation partially disrupts the reciprocal regulation between WSTF and its downstream effector GLYCTK.

Unsupervised clustering of the single-cell transcriptomes identified 17 coherent cell populations, which were categorized into three major lineages: immune, epithelial and stromal cells (**Supplementary Figure 1**). The expression profiling revealed that regions with high WSTF expression only partially overlapped with those enriched for GLYCTK, consistent with a cell-type dependent, partially mutually exclusive expression pattern (**Figures 9B, C**). GLYCTK expression was largely restricted to absorptive progenitors, with minor signals in B cells and CD4^+^ T cells, whereas WSTF was broadly expressed in malignant epithelial cells, absorptive progenitors, tumor stem cells and several immune subsets, including B cells, CD4^+^ and CD8^+^ T cells. These findings suggest that the apparent relationship between WSTF and GLYCTK may be influenced by distinct cellular compartments within the tumor microenvironment.

### GLYCTK-associated remodeling of the tumor immune microenvironment

**Figure 10:**
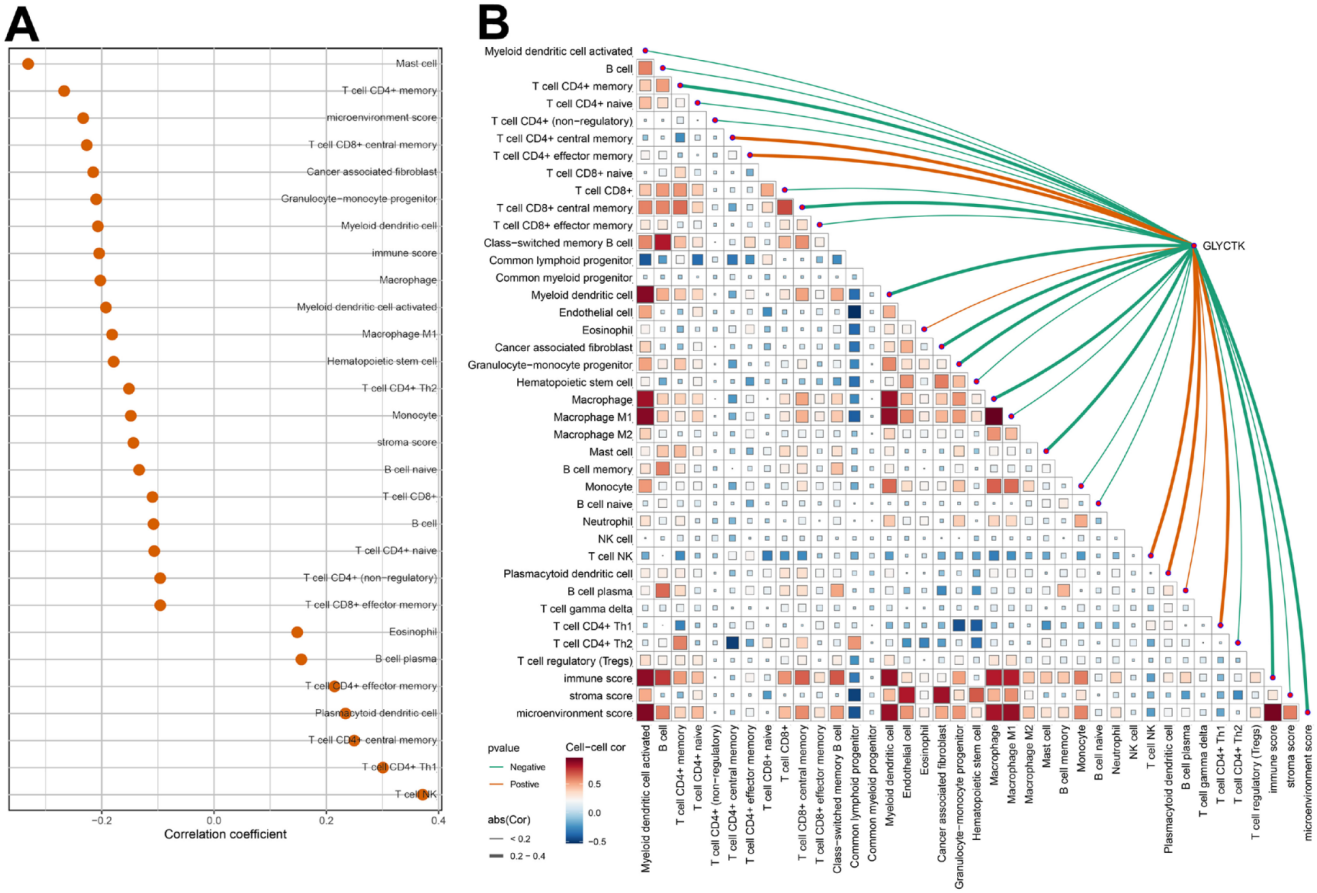
Association between GLYCTK expression and xCell-estimated immune cell infiltration in colorectal cancer. **(A)** Bubble plot showing spearman correlations between GLYCTK expression and infiltration scores of different cell types; only cell types with p < 0.05 are displayed. **(B)** LinkET correlation network. The heatmap shows pairwise correlations among different cell types, and the overlaying curves indicate significant correlations between GLYCTK expression and individual cell types.

Immune infiltration profiling based on xCell revealed that GLYCTK expression was associated with features of an immune-cold tumor microenvironment. Specifically, higher GLYCTK levels correlated negatively with multiple T-cell subsets, including CD4^+^ memory, CD4^+^ naive, CD8^+^, and CD8^+^ central memory T cells, as well as myeloid dendritic cells, macrophages, monocytes, and granulocyte-monocyte progenitors, indicating impaired antigen presentation and effector T-cell recruitment (**Figure 10**). In addition, GLYCTK expression showed significant negative correlations with the immune, stromal and microenvironment scores, further supporting a global reduction of non-malignant immune and stromal components in GLYCTK-high tumors. Conversely, GLYCTK was positively associated with NK cells, Th1 cells, plasmacytoid dendritic cells, and plasma B cells, together suggesting an immune landscape characterized by reduced overall infiltration but relative enrichment of specific immunoregulatory compartments (**Figure 10**).

As a potential upstream regulator, WSTF displayed broadly concordant but secondary associations in xCell, including negative correlations with effector memory CD4^+^ T cells and multiple myeloid lineages, supporting that WSTF may be associated with an immune-excluded environment along the same axis. These xCell-based associations were supported by CIBERSORT, where GLYCTK high expression coincided with increased regulatory T cells and plasma cells alongside reduced M1/M2 macrophages, and WSTF expression associated with altered B-cell subsets, resting NK cells, and diminished M2 macrophages (**Supplementary Figures 2**, **3**).

### Candidate drugs identification and molecular docking

**Figure 11:**
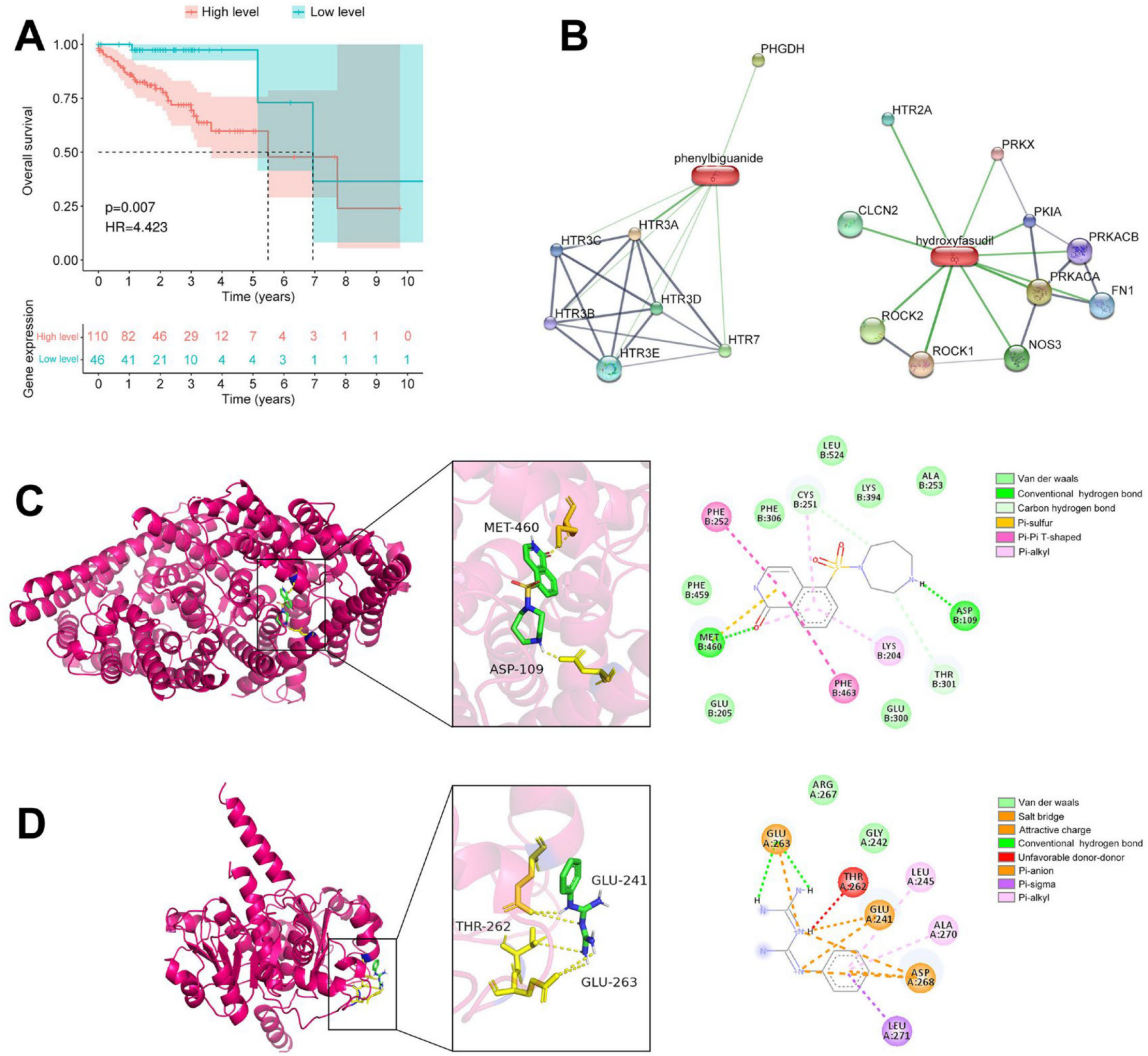
In silico candidate drug identification and molecular docking for colorectal cancer. **(A)** Kaplan-Meier survival curves stratified by GLYCTK and WSTF expression. **(B)** Drug-target interaction networks derived from the STITCH database. **(C)** Molecular docking model of hydroxyfasudil interacting with CLCN2. **(D)** Molecular docking model of phenylbiguanide interacting with PHGDH.

Survival analysis was conducted for GLYCTK and WSTF, with patients categorized into high- and low-risk groups based on the median expression (**Figure 11A**). Differential expression analysis between these groups identified 559 DEGs, with 307 genes upregulated and 252 downregulated in the high-risk group (**Supplementary Table 7**). These DEGs were submitted to the connectivity map (CMap) database, yielding two in silico candidate compounds with connectivity scores below -70, phenylbiguanide and hydroxyfasudil, which warrant future experimental evaluation for high-risk colorectal cancer patients (**Supplementary Table 8**).

Based on screening of the STITCH database, our study identified seventeen potential drug targets (**Figure 11B**). Of these, six genes displayed significant differential expression in the high-risk group. Subsequent survival analysis demonstrated that elevated expression of PHGDH and CLCN2 was associated with poorer patient survival, indicating their clinical relevance (**Supplementary Figure 4**). To validate binding affinities, molecular docking analyses were performed. Hydroxyfasudil docked to CLCN2 (PDB ID: 8TA2) with a binding free energy of -6.97 kcal/mol, interacting with residues such as Leu524, Met460 and Asp109 (**Figure 11C**). Phenylbiguanide docked to PHGDH (PDB ID: 6RJ6) with a binding free energy of -7.2 kcal/mol, forming interactions with residues including Glu241, Thr262 and Glu263 (**Figure 11D**). These results suggest that phenylbiguanide and hydroxyfasudil may interact with their predicted targets in silico, providing exploratory hypotheses for future experimental testing.

### GLYCTK expression and functional validation

**Figure 12:**
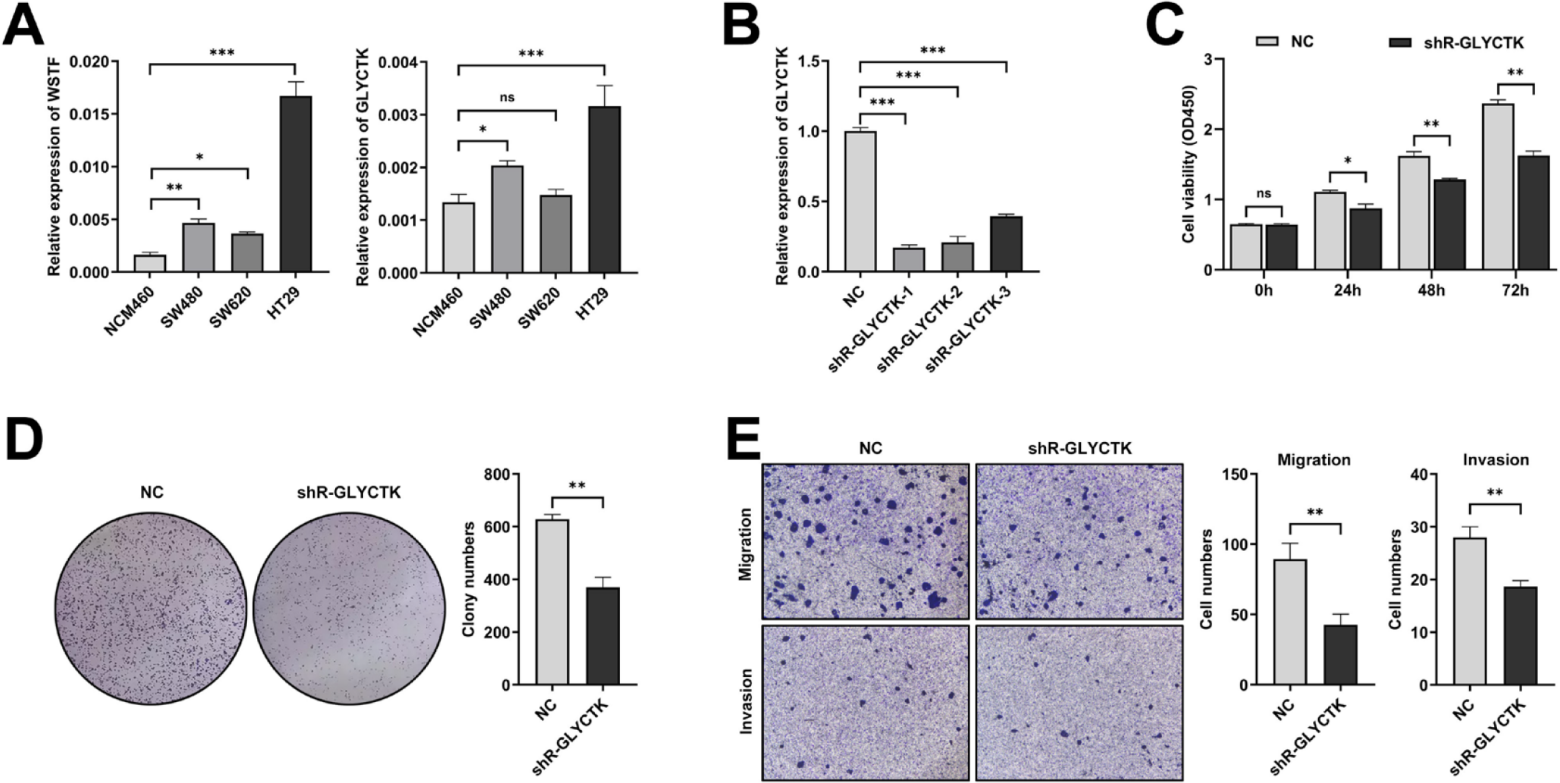
Effects of GLYCTK knockdown on CRC cell proliferation and migration. **(A)** Relative mRNA expression of WSTF and GLYCTK in CRC cell lines were quantified by RT-qPCR, using NCM460 (normal colorectal epithelial cells) as the control. Expression data were normalized to β-actin. **(B)** Efficiency of GLYCTK knockdown by three different shRNAs assessed by RT-qPCR in HT29 cells. Expression data were normalized to β-actin. **(C)** Cell viability measured by CCK-8 assay over 72 hours following GLYCTK knockdown compared to negative control (NC). **(D)** Colony formation assay in GLYCTK knockdown cell lines. **(E)** Transwell migration and invasion assays in GLYCTK knockdown cell lines, with statistical comparisons shown in right panel. *p < 0.05; **p < 0.01; ***p < 0.001; ns, no significance.

To investigate the biological function of GLYCTK, our study first assessed its expression alongside WSTF across multiple colorectal cell lines by RT-qPCR (**Figure 12A**). Both GLYCTK and WSTF showed significantly elevated expression in the cancer cell lines (SW480, SW620 and HT29) relative to the non-tumorigenic NCM460 cells. Among these cell lines, HT29 exhibited the highest transcript levels, thus it was selected for further functional studies. To identify an efficient GLYCTK silencing construct, three shRNA plasmids (shR-GLYCTK-1/-2/-3) were generated, and subsequently RT-qPCR showed that shR-GLYCTK-1 induced the strongest GLYCTK mRNA reduction in HT29 cells (**Figure 12B**). HT29 cells stably expressing shR-GLYCTK-1 were then selected to obtain three single-cell clones, among which clone 2 displayed the most pronounced and reproducible decrease in GLYCTK protein by Western blot and was used for subsequent functional experiments (**Supplementary Figure 5**). The impact of GLYCTK silencing on cell viability was measured by CCK8 assay, which revealed a significant decrease in proliferation observed at 24, 48 and 72 hours post-knockdown relative to negative controls (**Figure 12C**). Colony formation assays indicated that GLYCTK silencing significantly suppressed the clonogenic potential of HT29 cells (**Figure 12D**), similar to reduced proliferative capacity. Transwell assays demonstrated that GLYCTK knockdown significantly suppressed the migratory and invasive capacities of colorectal cancer cells (**Figure 12E**). Collectively, these findings suggest that GLYCTK may contribute to malignant phenotypes in HT29 cells by supporting proliferation, migration and invasion.

## Discussion

WSTF (BAZ1B), a regulator of chromatin remodeling and genome stability, emerges as a multifunctional driver of CRC progression (2). Combined transcriptomic and metabolomic analyses have shown that WSTF knockdown activates metal ion response, glucose metabolism and cell adhesion pathways, while suppressing ribosome biogenesis, genetic information processing and MYC-driven oncogenic programs. These results suggest that WSTF may support malignant phenotypes by being associated with growth-promoting transcriptional programs and adaptive metabolic changes. Genome-wide binding profiles further confirm WSTF as a transcription factor predominantly localized to promoter-proximal regions and recognizing GC-rich motifs, which enables the identification of GLYCTK as a candidate downstream target with functional relevance to CRC.

A key and seemingly contradictory finding is the strong inverse association between WSTF and GLYCTK expression. In bulk transcriptomic data, GLYCTK expression was negatively correlated with WSTF in normal colon, suggesting an inverse expression relationship under physiological conditions. Similarly, WSTF knockdown led to a significant increase in GLYCTK expression, supporting a model in which WSTF may be associated with transcriptional repression of GLYCTK in CRC cells.

Single-cell transcriptomic profiling further refines this interpretation by revealing a cell-type dependent, partially exclusive pattern. GLYCTK expression is largely restricted to absorptive progenitors, with minor signals in B cells and CD4^+^ T cells. In contrast, WSTF is widely expressed in malignant epithelial cells, absorptive progenitors, tumor stem cells and multiple immune subsets. This distribution indicates that bulk-level correlations arise from two main sources. One is the intra-cellular regulation of GLYCTK by WSTF within shared cell subsets. The other is inter-cellular composition, driven by their enrichment in different cell lineages. In normal colon, where the cellular makeup is relatively stable, WSTF-dependent repression is likely the dominant influence and produces a strong negative correlation. In tumors, however, clonal heterogeneity, lineage shifts and immune remodeling change the proportions of cell types along this regulatory axis, which weakens, but does not completely abolish, the inverse relationship.

SMR analysis provides additional mechanistic insight into the WSTF-GLYCTK axis. Two CpG sites (cg11209338 and cg01758942) showed strong causal relationships with both GLYCTK transcription and CRC susceptibility. higher methylation at these sites was negatively associated with GLYCTK expression but positively associated with CRC susceptibility, whereas GLYCTK expression itself was negatively associated with CRC susceptibility. These data suggest that methylation-mediated suppression of GLYCTK contributes to tumor initiation, and that maintaining adequate GLYCTK expression may be protective at the level of disease susceptibility.

Functional assays show that GLYCTK promotes CRC cell proliferation, colony formation, migration and invasion, supporting a pro-tumorigenic role. Importantly, the SMR analysis reflects susceptibility at the population level, whereas the cell-based assays capture tumor-cell behavior after malignant transformation. These results appear different from the SMR findings, which suggest that higher GLYCTK expression may be associated with lower CRC risk. This apparent discrepancy may be explained by considering that the role of GLYCTK depends on disease stage and context. In the pre-malignant setting, sufficient GLYCTK activity may help maintain metabolic homeostasis in D-glycerate-related pathways and limit chronic metabolic or genotoxic stress, thereby reducing long-term CRC susceptibility. After malignant transformation, however, the same metabolic pathway can be re-purposed by cancer cells to support anabolic demands and more aggressive behavior. Thus, GLYCTK may act as a context-dependent effector, protecting against tumor initiation when appropriately expressed in normal epithelium, yet facilitating tumor progression once it is co-opted by malignant clones.

The negative correlation between WSTF and GLYCTK becomes weaker in tumors relative to normal tissues, suggesting that malignant transformation disrupts the tight reciprocal pattern. This weakening fits a model in which oncogenic WSTF acts within a strongly reshaped regulatory environment, while GLYCTK becomes increasingly governed by DNA methylation, cell-of-origin composition and tumor microenvironmental cues. Under these conditions, the WSTF-GLYCTK relationship becomes more variable and context-dependent, with some tumor cell populations preserving a repressive WSTF-GLYCTK link and others escaping it through epigenetic and transcriptional reprogramming.

The immune microenvironment analyses extend the WSTF-GLYCTK axis into immune regulation. In computational immune infiltration analyses, High GLYCTK expression was associated with an immune-cold phenotype: reduced infiltration of T-cell subsets, macrophages, dendritic cells and monocytes, along with lower stromal and microenvironment scores, but relative enrichment of NK cells, Th1 cells, plasmacytoid dendritic cells and plasma B cells (37). WSTF expression shows a broadly similar but non-identical pattern, including negative correlations with effector memory CD4^+^ T cells and multiple myeloid populations and positive associations with mast cells and naive B cells. Taken together with CIBERSORT analysis, these results suggest that WSTF may contribute to a metabolically active, immune-excluded tumor niche, whereas GLYCTK contributes to a more profoundly immunosuppressed microenvironment with altered B-cell and regulatory T-cell activity.

Genome-wide ChIP-seq revealed that WSTF preferentially binds proximal promoter regions and recognizes GC-rich motifs. Within the GLYCTK regulatory region, three distinct clusters of WSTF motifs in the promoter and 5’ UTR contain a conserved CCGCC core and repetitive GC elements, suggesting recruitment through GC-rich chromatin and potential modulation of transcription initiation. The 5’ UTR motif may influence RNA secondary structure and post-initiation regulation (13). Taken together, the proposed WSTF-GLYCTK model can be summarized as follows. WSTF binding to GC-rich motifs within the GLYCTK locus may be associated with chromatin-level regulation of GLYCTK, while SMR-identified methylation sites may represent additional upstream regulatory signals. Methylation at cg11209338 and cg01758942 causally suppresses GLYCTK expression but increases CRC risk, whereas higher GLYCTK expression is causally linked to reduced cancer risk. In this model, WSTF and methylation may together contribute to a repressive regulatory state at the GLYCTK locus in specific cellular contexts, thereby contributing to the overall inverse association between WSTF activity and GLYCTK levels in colorectal tissues.

In conclusion, this study provides multi-omics evidence supporting a role for WSTF in colorectal cancer progression through transcriptional and epigenetic-associated regulation of metabolic adaptation, with GLYCTK emerging as a candidate effector. These findings broaden the understanding of WSTF-associated regulatory networks in CRC and identify candidate molecules for future mechanistic and translational studies. This study is limited by the absence of direct mechanistic assays (including ChIP-qPCR, promoter methylation analysis after WSTF perturbation, and chromatin-regulator recruitment experiments), which will be important in future work to confirm the proposed epigenetic mechanism. In addition, the immune-related findings are based solely on xCell, CIBERSORT and scRNA-seq computational analyses without tissue-level or functional immune validation, which will be required to determine whether these associations reflect causal immunological mechanisms.

## Data Availability

The datasets generated for the WSTF-related analyses in this study have been deposited in the NCBI Sequence Read Archive (SRA) and the BioSample repository (accession numbers PRJNA1454275, https://www.ncbi.nlm.nih.gov/bioproject/PRJNA1454275). Additional datasets analyzed in this study were obtained from publicly available repositories, the corresponding database names and accession numbers are provided in the Methods section.
