# Supplementary material for: WSTF-associated regulation of GLYCTK and metabolic adaptation in colorectal cancer
Source: Front Immunol. 2026 Apr 23;17:1819148. doi: 10.3389/fimmu.2026.1819148 (PMC13150450; doi:10.3389/fimmu.2026.1819148)
Supplement: Supplementary file 1 [file DataSheet1.docx]

**
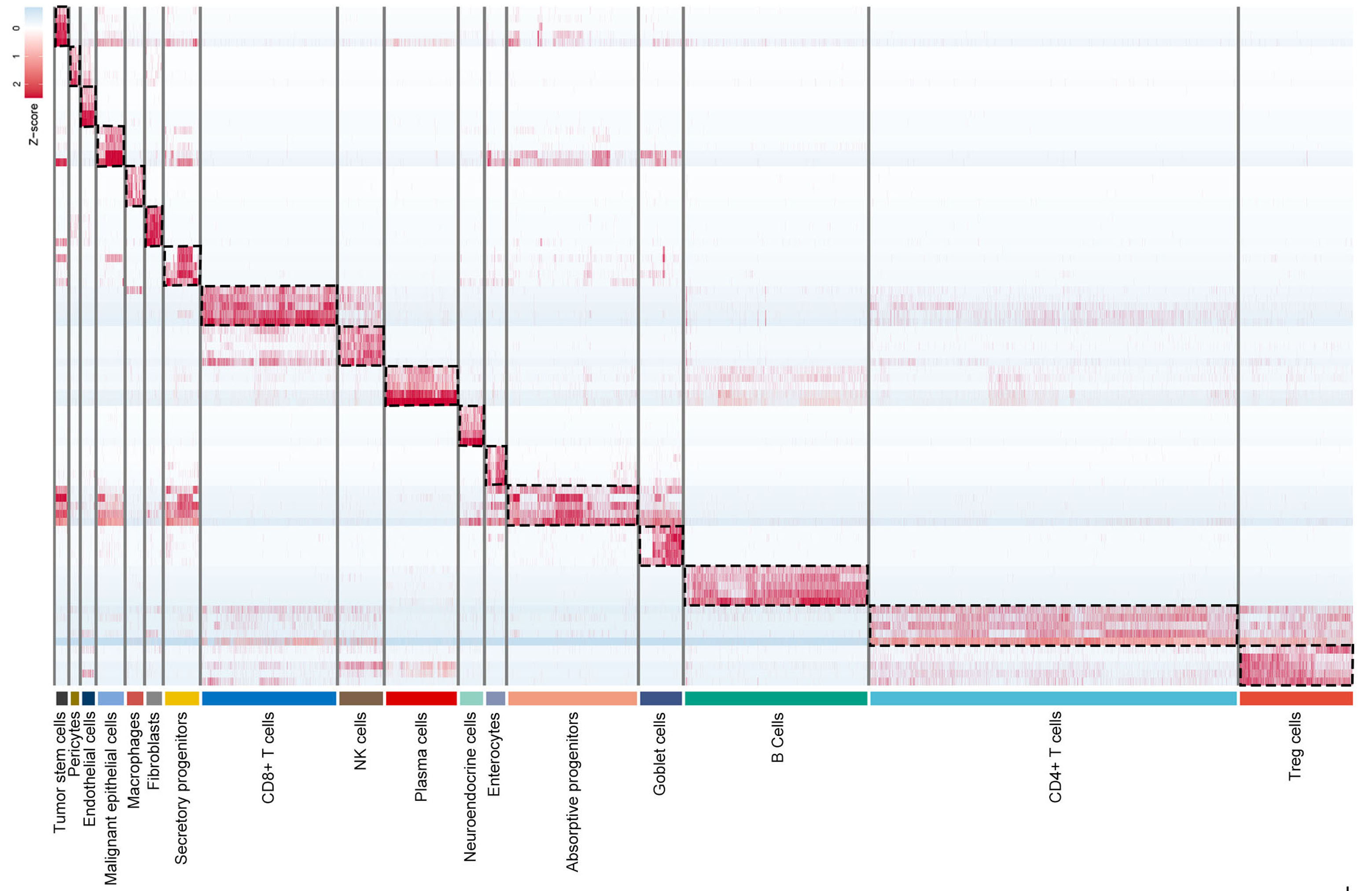
**

**SUPPLEMENTARY FIGURE 1**

Heatmap of top 5 differentially expressed marker genes among the defined cell types.

**
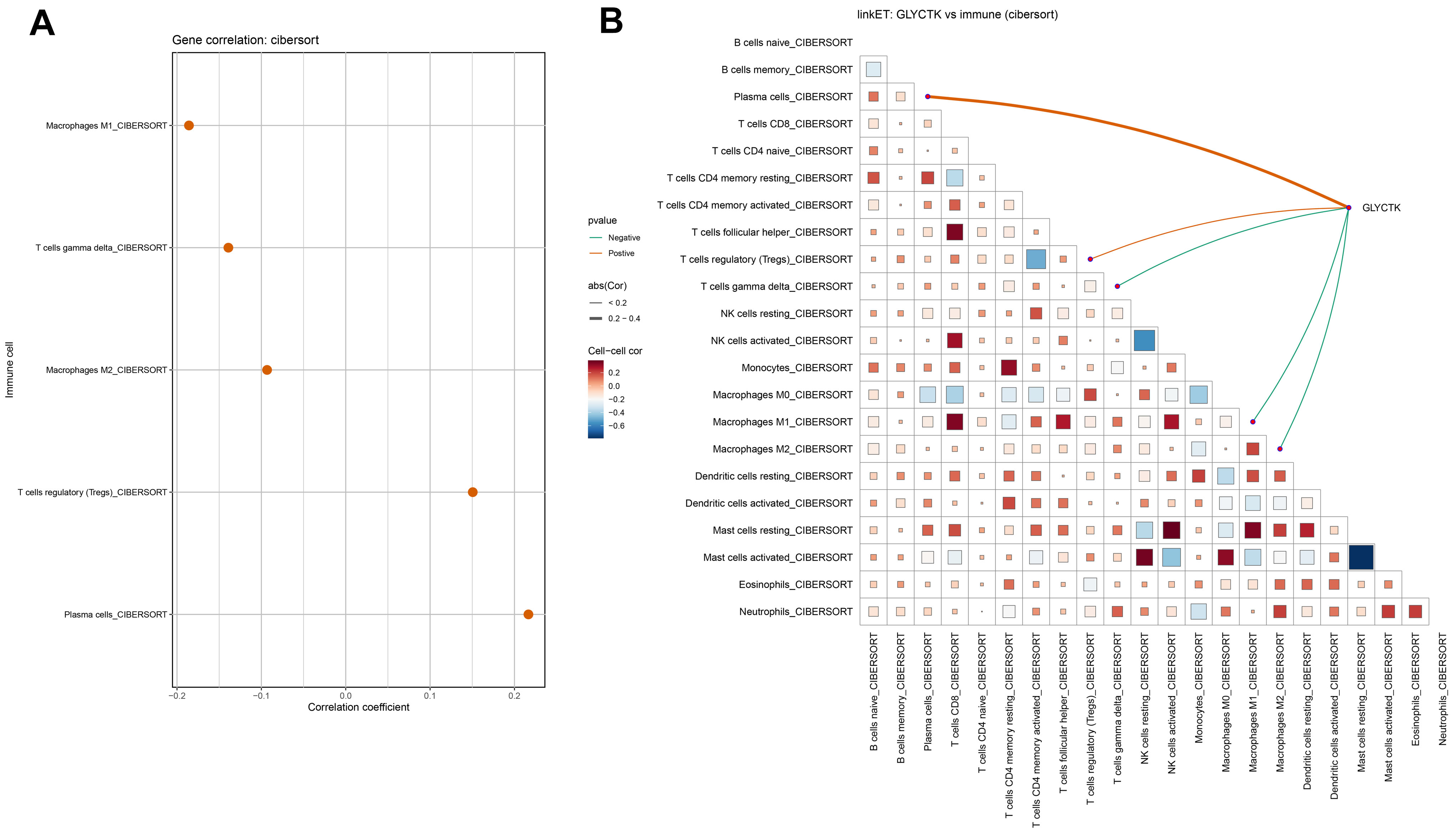
**

**SUPPLEMENTARY FIGURE 2**

Correlation between GLYCTK expression and immune cell infiltration. **(A)** Bubble plot showing spearman correlations (CIBERSORT). **(B)** LinkET correlation network (CIBERSORT).

**
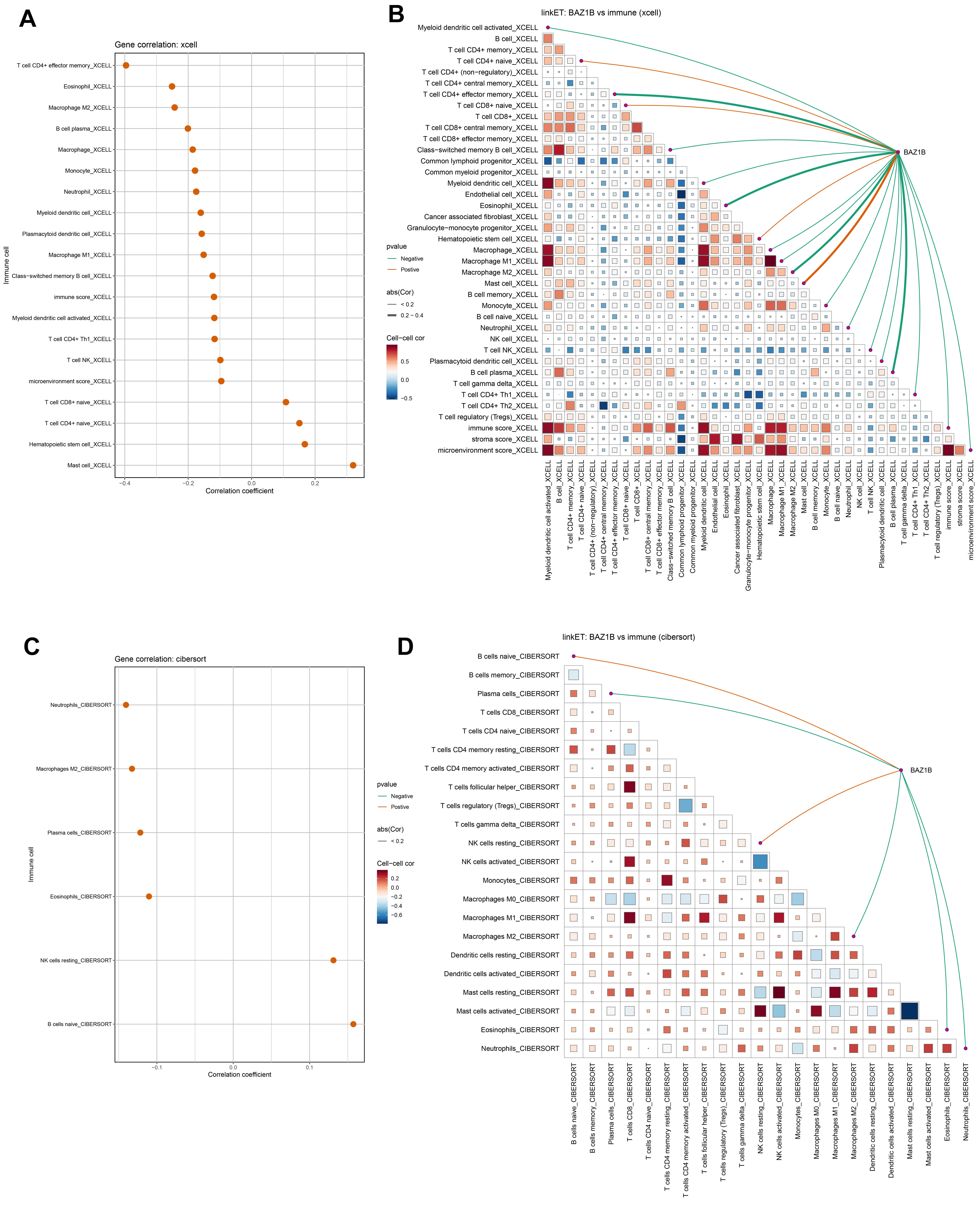
**

**SUPPLEMENTARY FIGURE 3**

Correlation between WSTF expression and immune cell infiltration. **(A)** Bubble plot showing spearman correlations (xCell). **(B)** LinkET correlation network (xCell). **(C)** Bubble plot showing spearman correlations (CIBERSORT). **(D)** LinkET correlation network (CIBERSORT).

**
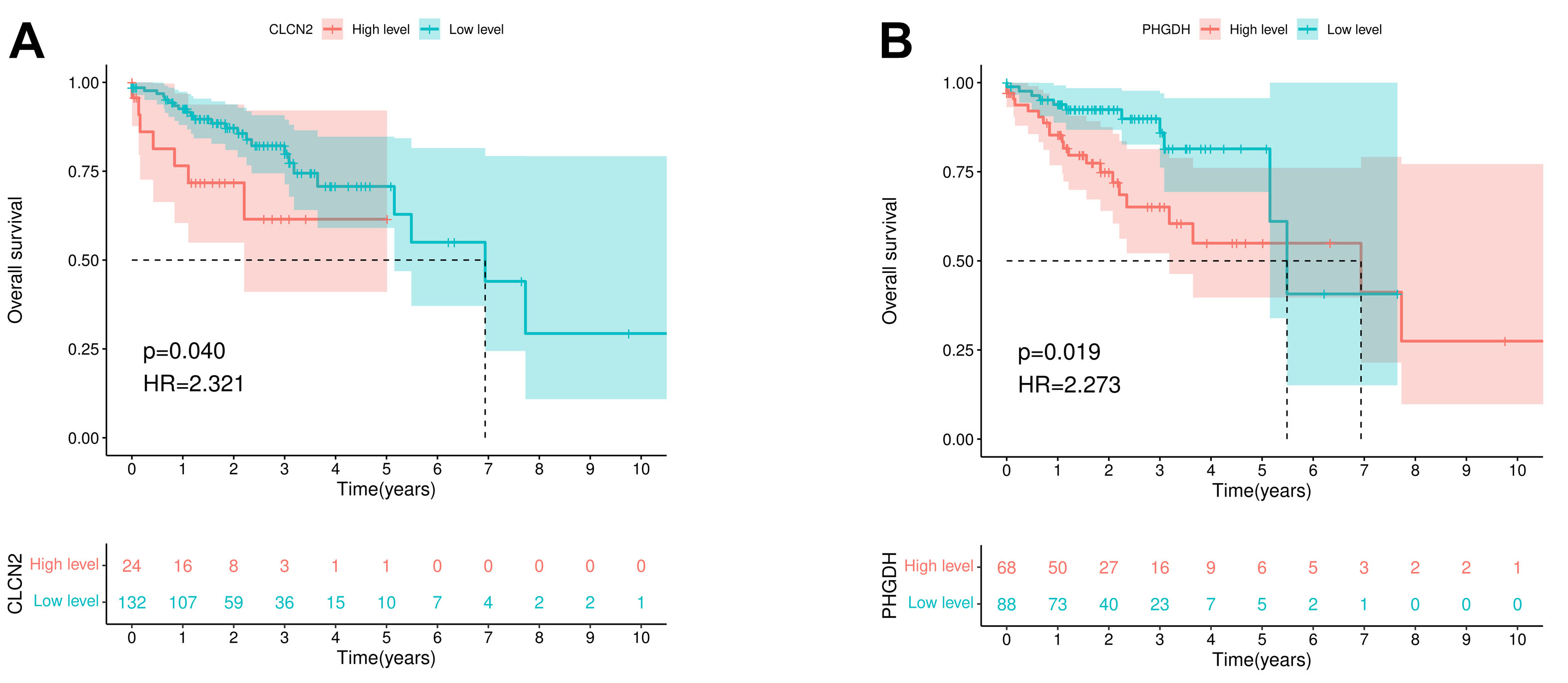
**

**SUPPLEMENTARY FIGURE 4**

Survival analysis of CLCN2 **(A)** and PHGDH **(B)**.

**
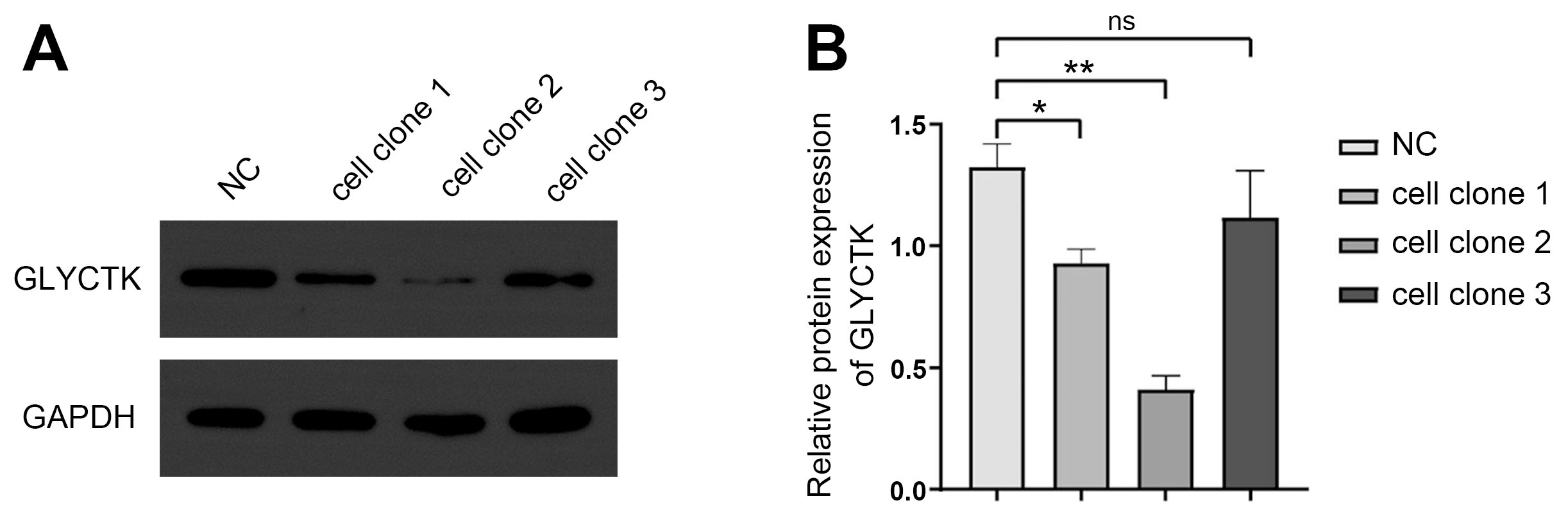
**

**SUPPLEMENTARY FIGURE 5**

GLYCTK knockdown validation in HT29 cells. **(A)** Western blot analysis of GLYCTK in negative control (NC) and three single‑cell clones, GAPDH as loading control. **(B)** Quantification of GLYCTK protein expression levels relative to NC (mean ± SD, ns, *P < 0.05, **P < 0.01).
